# Passage of Time Judgments Is Relative to Temporal Expectation

**DOI:** 10.3389/fpsyg.2017.00187

**Published:** 2017-02-14

**Authors:** Ryosuke Tanaka, Yuko Yotsumoto

**Affiliations:** Department of Life Sciences, The University of TokyoTokyo, Japan

**Keywords:** time, time perception, passage of time, expectation, temporal expectation

## Abstract

Time seems to pass quickly sometimes or slowly at other times. While this belief is prevalent, the psychological bases of such judgments on speed of time have remained unclear. In this study, we tested following two hypotheses: (1) the passage of time judgment (POTJ) is a function of the discrepancy between felt duration and temporal expectation of events and (2) POTJ is based on two distinct components: *post hoc* comparison of expected and felt durations and online anticipation of the end of an event. In four experiments, participants engaged in N-back tasks for several minutes and rated their POTJ during the tasks. Their temporal expectations were manipulated by providing them with false instructions on task durations. The results consistently supported the hypotheses and confirmed the idea that temporal expectation plays an important role in POTJ. In addition, the current findings might explain our daily temporal experiences such as “time flies when you are having fun.”

## Introduction

Although the past several decades have seen significant accumulation of experimental psychological studies on human time perception, a considerable portion of those works were dedicated to estimation of duration (Fraisse, 1984; Buhusi and Meck, 2005). One prevalent temporal experience that seems different from duration estimation is the change of the speed of time passage. For example, statements such as “time flies when you are having fun” or “time drags at work” seem quite common. Such statements on the speed of time, or passage of time judgments (POTJ), have recently started to attract the interest of researchers (Ogden et al., 2011; Wearden, 2012, 2015; Droit-Volet and Wearden, 2016), although their cognitive mechanisms are still unclear.

Of particular interest when examining POTJ are the relationships between POTJ and duration estimation. It is well known that subjective duration estimation dilates or contracts under influence of various non-temporal stimuli features (Kanai et al., 2006; Pariyadath and Eagleman, 2007) and the observer’s psychological state (Block et al., 2010). At first glance, such duration distortion seems similar to quickening or slowing of POTJ. Actually, expressions such as “time flies” or “time drags” are frequently used in research articles on the distortion of duration estimation. However, there exists critical differences between duration distortion and POTJ change, that is, subjective time dilation or contraction itself does not accompany any feeling that time is passing by quickly or slowly (Wearden, 2015). This means that people do not judge “speed of time,” directly based on “clock speed” which is hypothesized in internal clock models of duration perception (Treisman, 1963; Buhusi and Meck, 2005; Gorea, 2011).

So, what exactly do we mean when we say it is as if time is flying or dragging? An important point is that, unlike original concept of speed, “speed of time” is not a physical quantity and it is impossible to think of “absolute speed of time” without any comparison. For example, when physicists say that time slows in a fast moving rocket in the context of the theory of relativity, it means that an event (e.g., a tick of a clock) in the rocket looks longer to an observer on the stationary ground compared to the same event on the ground. Also in the psychological context, it seems that our remarks that time seems passing by fast or slowly always connotes that it seems “faster” or “slower” than usual.

Based on the observations above, we hypothesized that POTJ is essentially based on relative discrepancy between expected and felt durations of an event. More specifically, if a certain event ends sooner than expected, quick POTJ should arise, and if an event lasts longer than expected, slow POTJ should arise. This hypothesis is followed by certain asymmetry between fast and slow POTJs. That is, while sensation that an event was “shorter than expected” must always be preceded by the end of the event, one can notice that the task is lasting “longer than expected” even during the event. Therefore, we additionally hypothesized that there are two distinct components that POTJ is based on: (1) *post hoc* comparison between the felt duration of an event and the expectation of how long it should feel like and (2) online anticipation during an event concerning how likely the event is to end immediately. Online anticipation of the end of the event has been discussed previously as hazard functions (Nobre et al., 2007) or posterior time information (Mo, 1990; Laflamme et al., 2015).

Note that our hypotheses incorporating online anticipation require following premises: (1) participants have certain mental standards about how long x-minutes should feel like. This idea is directly supported by that one can verbally estimate or produce durations (Zakay, 1993). Such standards are assumed to work as prior temporal information (Mo, 1990; Laflamme et al., 2015), which enables temporal anticipation of the end of an event. (2) Participants are implicitly keeping track of time prospectively even when they are not asked to do so. This assumption is pertinent to a recent model which states that prospective duration estimation is automatically initiated when importance of time information for a person (or temporal relevance) is reasonably high (Zakay, 2015) or studies that showed implicit timing shares characteristics with prospective duration estimation (Nobre et al., 2007; Piras and Coull, 2011).

**FIGURE 1:**
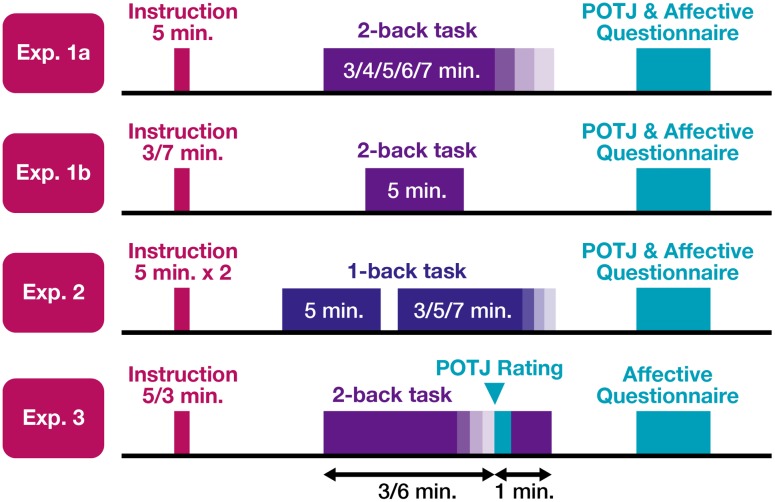
**The schematic of the experiments.** In all experiments, participants received instructions on how long the task sessions would take, which were sometimes false and sometimes true. They then underwent one or two sessions of N-back tasks, whose durations ranged from 3 to 7 min. In Experiments 1a, 1b, and 2, participants completed a questionnaire that asked their passage of time judgment (POTJ) and affective states. In Experiment 3, to remove the *post hoc* component on POTJ, participants were unexpectedly asked to rate their POTJ during the 2-back task sessions.

To test these two hypotheses, the discrepancies between the actual and expected durations during which participants engage in certain tasks were manipulated by giving them false instructions concerning durations. Experiments 1a, 1b, and 2 sought to test the former hypothesis that POTJ is a function of the difference between temporal expectation and implicit duration estimation. Experiment 3 was conducted to show the asymmetry of quick and slow POTJ. The schematic of the all four experiments is **Figure 1**.

## Experiment 1a

In Experiment 1a, the actual durations that participants engaged in a task were manipulated, while instructions on how long the task would take were kept constant.

### Methods

#### Participants

Fifty-six males and forty-two females (age range = 18-50 years, mean age = 19.58 ± 3.52) participated in the experiment. No participants were excluded. All participants had normal or corrected to normal vision. All participants gave written informed consent for their participation in the experimental protocol, which was approved by the institutional review boards of The University of Tokyo. The sample sizes were decided in an *a priori* manner since sizes of fixed and random effects were unpredictable. The numbers were comparable with the study which recruited a similar manipulation as ours (Sackett et al., 2010).

#### Apparatus

The experimental programs were run on Matlab R2015b (The MathWorks, Inc., Natic, MA, USA) with a PsychToolbox 3.0 extension (Kleiner et al., 2007). All visual stimuli were presented on 17 in. CRT monitor (CPD-E230, Sony, Tokyo, Japan).

#### Task

In the experiment, participants performed a so-called 2-back task. During the task, white one-digit numbers (i.e., 0–9) were presented sequentially against the black background. Participants were required to click the left mouse button if the presented number matched with the number from the number two earlier steps in the sequence and the right button if it mismatched. Immediately following the response, participants visually received feedback (a green circle for correct; a red X for wrong; overlaid on the number until it disappeared). The order of the numbers was randomly determined so that the probability of match and mismatch were the same. Each number was presented for 1.5 s, and there were 0.5 s blank intervals between numbers. The numbers extended approximately 2 cm × 3 cm and the viewing distance was approximately 60 cm, although it was not fixed.

#### Questionnaire

We created a questionnaire that assessed participants’ POTJ along with other affective variables that have been reported to correlate with POTJ in previous studies. The questionnaire employed visual analog scales (VAS). The scales were 100 mm long in the horizontal axis. Participants marked the position that best corresponded to their internal states with a pen. The positions of the marks were quantified in the step of 1 mm so that each variable took an integer value between 0 and 100. Questions were as follows: (1) During the task, how fast did the time seem to be passing by? (POTJ) (2) During the task, how happy did you feel? (Positive mood) (3) During the task, how sad did you feel? (Negative mood) (4) During the task, how aroused/awake were you? (Arousal) (5) During the task, how relaxed were you? (Relaxation) (6) During the task, how bored were you? (Boredom) (7) How difficult was the task for you? (Task difficulty) Note that actual questions presented in the experiments are in Japanese language.

#### Procedure

Each participant was randomly assigned to one of five duration conditions: 3, 4, 5, 6, and 7 min. The number of participants assigned to each condition were 18, 21, 18, 22, and 19, respectively. Prior to the actual task session, all participants received instructions that the task would be 5 min long, regardless of what duration condition the participant was assigned. Similar procedures were recruited in several previous studies (Sackett et al., 2010; Park et al., 2016). Participants first learned the rule of the 2-back task in a brief (usually less than 1 min) practice session on a laptop. Participants then entered a dark soundproof chamber and performed the 2-back task for the certain duration they were assigned to. Participants were instructed to remove their watches and phones before entering the chamber “in order to focus on the task,” which was actually intended to deprive the participants of external temporal information. After performing the task, participants filled in the POTJ questionnaire. It was only after the task session that participants were told that they were going to be asked about time.

#### Analysis

Since POTJ rating scores were discrete values with a finite range, we first tried to recruit a binomial distribution to model the scores. However, because the scores yielded much larger variance than can be explained by a binomial distribution alone, we decided to use a generalized linear mixed model (GLMM) that posits a normally distributed random effect (Bolker et al., 2009), which corresponds to unobserved individual differences. Therefore, our statistical model was as follows:

$$\begin{array}{c} \text{POTJ}∼\text{Binomial}\left(q_{i},100\right)\\ \text{where logit}\left(q_{i}\right)=\beta_{0}+\beta_{1}X_{1}+\beta_{2}X_{2}+....+r_{i}\\ r_{i}∼N\left(0,\sigma^{2}\right) \end{array}$$

(X*_k_* is a questionnaire scores or a task performance and β*_k_* is its regression coefficient. *r_i_* stands for the random effect across participants.)

All analyses were conducted on R with the “glmmML” extension (Broström and Holmberg, 2011). Raw data for all experiments can be found at http://webpark1842.sakura.ne.jp/data/POTJ/.

### Results

#### Task Performance

The mean correct rate was 92.97%, and all participants performed better than 75%. The mean response time was 620.1 ± 123.7 ms. There was no difference in the task performance across the conditions.

#### Statistical Modeling

**FIGURE 2:**
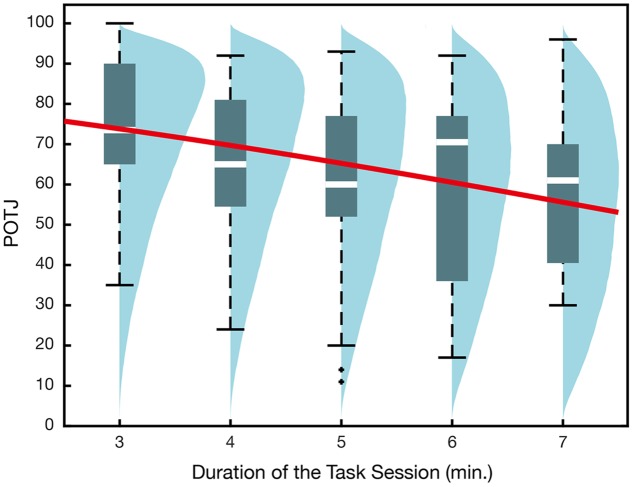
**Passage of time judgment in Experiment 1a.** The x-axis corresponds to the durations that participants actually worked on the 2-back task. All participants were instructed that the task session would take 5 min. The overlaid regression curve indicates mean q_i_ obtained in GLMM recruiting actual duration only as the explanatory variable. Blue density plots are simulated POTJ distribution density incorporating random effects.

The GLMM using actual duration alone as an explanatory variable yielded negative regression coefficient whose 95% confidence interval did not include 0 (**Table 1**, **Figure 2**). That is, the longer the task durations were compared to the initial instructions, the slower the participants tended to rate their POTJ. The model selected based on the Akaike information criterion (AIC) indicated that higher sadness and subjective task difficulty, as well as longer task durations, led to slower POTJ (**Table 2**). The valence and significance of regression coefficients remained almost the same when we used linear modeling assuming normality instead of binomial GLMM (Intercept: β = 87, *p* < 10^-15^; Duration: β = -3.4, *p* = 0.05; Sadness: β = -0.17, *p* = 0.08; Boredom: β = -0.17, *p* = 0.07). Additional inspection revealed some significant correlations among the variables (sadness and correct response rate, *r* = -0.30, *p* = 0.03; relax and difficulty, *r* = -0.34, *p* = 0.01; difficulty and correct response rate, *r* = -0.41, *p* = 5.7 × 10^-4^; correct response rate and reaction time, *r* = -0.41, *p* = 5.7 × 10^-4^). Although several studies have reported sex differences in time estimation (Glicksohn and Hadad, 2011; Sanders and Sinclair, 2011), adding sex as an explanatory variable did not improve AIC. All *p*-values were false discovery rate corrected.

**Table 1:** Generalized linear mixed model (GLMM) recruiting actual duration only as an explanatory variable for the results of Experiment 1a.

	β	*SE*	*Z*	P (>|Z|)
Intercept	1.6	0.43	3.9	1.2 × 10^-4^
Duration (min.)	-0.20	0.08	-2.5	1.3 × 10^-2^

	**σ**	***SE***		

Random effect	1.1	0.08		


*AIC = 412, conditional *R*^*2*^ = 0.28. All *R*^*2*^-values were calculated according to Nakagawa and Schielzeth (2013). SE, standard error; Z, Wald statistic; P(>|Z|) = probability that a more extreme estimation of β than observed is obtained given that true β = 0; GLMM, generalized linear mixed model.*

**Table 2:** Generalized linear mixed model (GLMM) selected based on AIC for the results of Experiment 1a.

	β	*SE*	*Z*	P (>|Z|)
Intercept	2.0	0.43	4.7	2.7 × 10^-6^
Duration (min.)	-0.19	0.08	-2.5	1.4 × 10^-2^
Sadness	-9.5 × 10^-3^	4.9 × 10^-3^	-1.9	5.4 × 10^-2^
Boredom	-8.5 × 10^-3^	4.4 × 10^-3^	-1.9	5.3 × 10^-2^

	**σ**	***SE***		

Random effect	1.0	0.08		


*AIC = 407.7; conditional *R*^*2*^ = 0.28; SE, standard error; GLMM, generalized linear mixed model; Z = Wald statistic; P(>|Z|) = probability that a more extreme estimation of β than observed is obtained given that true β = 0; AIC, Akaike information criterion.*

## Experiment 1b

In Experiment 1b, the instruction about how long the task would last was manipulated, while the actual duration that participants performed the task was set constant. This manipulation was intended to exclude the possibility that POTJ simply correlated with the task durations, regardless of expectation.

### Methods

Twenty males and eleven females (age range = 18-21 years, mean age = 19.03 ± 0.71) participated in the experiment. None of the participants participated in Experiment 1a. No participants were excluded. The methods were basically identical to Experiment 1a except instructed and actual durations of the tasks. Each participant was assigned to either a 3 or a 7 min condition. The numbers of participants assigned each condition were 16 and 15, respectively. Participants received false instructions that the task was going to last either 3 or 7 min according to the condition they were assigned. The actual task duration was fixed to 5 min.

### Results

#### Task Performance

The mean correct rate was 94.93%, and all participants performed better than 85%. The mean response time was 620.9 ± 117.4 ms. There was no difference in the task performance between the conditions.

#### Statistical Modeling

**FIGURE 3:**
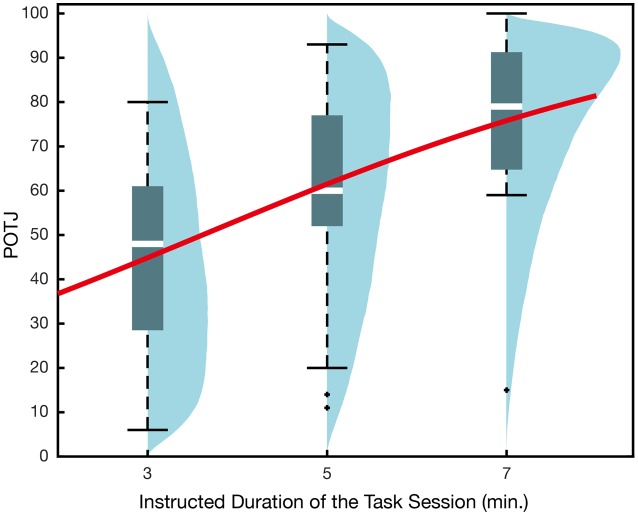
**Passage of time judgment in Experiment 1b.** The *x*-axis corresponds to the instructed durations of the task sessions. Every participant actually underwent a 5-min session of the 2-back task. The overlaid regression curve indicates mean q_i_ obtained in GLMM recruiting actual duration only as the explanatory variable. Blue density plots are simulated POTJ distribution density incorporating random effects.

The data from Experiment 1b and the data from the 5-min condition in Experiment 1a (*N* = 18) were merged and then submitted to the analyses. The GLMM using instructed duration alone as explanatory variable yielded positive regression coefficient whose 95% confidence interval did not include 0 (**Table 3**, **Figure 3**). In other words, the longer the instructed durations of the task sessions, the faster the participants tended to rate their POTJ. The model selected based on AIC indicated that the higher arousal and boredom predicted slower POTJ, while the higher correct response rate as well as the longer instructed durations led to faster POTJ (**Table 4**). The valence and significance of regression coefficients remained almost the same when we used linear modeling assuming normality instead of binomial GLMM (Intercept: β = -130, *p* = 0.09; Duration: β = 6.6, *p* = 6.2 × 10^-3^; Arousal: β = -0.20, *p* = 0.16; Boredom: β = -0.36, *p* = 8.8 × 10^-3^; Correct Rate: β = 190, *p* = 0.02). No significant correlations among the variables were found and adding sex as an explanatory variable did not improve AIC.

**Table 3:** Generalized linear mixed model recruiting instructed duration only as an explanatory variable for the results of Experiment 1b.

	β	*SE*	*Z*	P (>|Z|)
Intercept	-1.6	0.57	-2.7	6.8 × 10^-3^
Duration (min.)	0.40	0.11	3.7	2.6 × 10^-4^

	**σ**	***SE***		

Random effect	1.2	0.12		


*AIC = 215.2, conditional *R*^*2*^ = 0.36; SE, standard error; Z = Wald statistic; P(>|Z|) = probability that a more extreme estimation of β than observed is obtained given that true β = 0; GLMM, generalized linear mixed model.*

**Table 4:** Generalized linear mixed model selected based on AIC for the results of Experiment 1b.

	β	*SE*	*Z*	P (>|Z|)
Intercept	-9.6	3.6	-2.6	8.5 × 10^-#^
Duration (min.)	0.37	0.10	3.7	2.0 × 10^-4^
Arousal	-0.01	7.5 × 10^-3^	-1.6	1.2 × 10^-1^
Boredom	-0.02	6.1 × 10^-3^	-3.0	2.7 × 10^-3^
Correct rate	10	3.8	2.7	7.6 × 10^-3^

	**σ**	***SE***		

Random effect	1.0	0.11		


*AIC = 208.3; conditional *R*^*2*^ = 0.36; SE, standard error; Z = Wald statistic; P(>|Z|) = probability that a more extreme estimation of β than observed is obtained given that true β = 0; GLMM, generalized linear mixed model; AIC, Akaike information criterion.*

### Discussion

Experiments 1a and 1b were intended to test the hypothesis that POTJ was proportional to the difference between expected and actual duration. As hypothesized, the longer the task duration relative to prior instruction was, the slower POTJ was predicted. Several affective variables also predicted slower or quicker POTJ, partly replicating a previous study (Droit-Volet and Wearden, 2016). Among them, boredom consistently predicted slower POTJ in both analyses, which is in accord with a previous suggestion that boredom, accompanies felt time elongation or slowing (Zakay, 2014). However, note that the analyses on relationships between POTJ and affective variables in Experiments 1a and 1b were not independent, as we reanalyzed some data.

It is worth noting that mean POTJ larger than 50, that is, quicker than usual, was observed even in the 7-min instruction condition in Experiment 1a. This was likely due to relative underestimation of task duration that resulted from deprivation of attention toward time by the 2-back task, which is highly working-memory demanding (Zakay, 1998; Block et al., 2010).

The GLMM analyses conducted so far yielded a normally distributed random effect with a standard deviation around 1.0 and a relatively low *R*^2^ value. One plausible source of this relatively large unobserved individual difference is the variance of implicit duration estimation, which is likely to follow the scalar property (Buhusi and Meck, 2005). Another probable scenario is that the expectations on how long certain minutes feel like varied across participants. For example, participants who regularly engage into cognitively demanding 5 min tasks (e.g., video games) might have had relatively shorter memory representations of 5 min, while some others who are used to vacant 5 min (e.g., waiting for foods to be microwaved) might have had longer representations of 5 min. To discern these two possibilities, we conducted Experiment 2.

## Experiment 2

Experiment 2 was conducted to narrow down possible sources of overdispersion that were observed in Experiments 1a and 1b. In Experiment 2, participants performed two successive alleged 5-min sessions of the task. The duration of the first session was actually fixed to 5 min, while the duration of the second session was either 3, 5 or 7 min. The first session was installed in order to provide participants with a better idea of how long 5 min in the specific context (i.e., 1-back task in a dark chamber) feel like. Here, we expected that, if the overdispersion of POTJ resulted from individual differences of mean temporal expectation, making participants experience 5-min beforehand should minimize such biases and thus mitigate the overdispersion.

### Methods

Thirty-one male and twenty-nine female participants (age range = 18-22 years, mean age = 19.05 ± 0.95) took part in the experiment. None of the participants participated in Experiments 1a or 1b. No participants were excluded. The apparatus was the same as in Experiments 1a and 1b. In Experiment 2, participants performed a 1-back task, instead of a 2-back task. The temporal and spatial configurations of the stimuli were identical to Experiments 1a and 1b.

#### Procedure

Each participant was assigned to one of three duration conditions: 3-, 5-, and 7-min conditions. Twenty participants were assigned to each condition. After learning the rule of the task in a short practice session, participants underwent two task sessions inside a dark soundproof chamber. The duration of the first session was fixed to 5 min, whereas the duration of the second session was varied according to the condition the participant was assigned. Participants received the instruction that the durations of both sessions would be 5 min, regardless of the actual durations. Between the sessions, participants took a brief rest of several minutes outside the chamber. After completing the second session, participants completed the POTJ questionnaire.

### Results

#### Task Performance

The mean correct rates were 97.62 and 97.45% for the first and second sessions, respectively. All participants performed better than 90% in both conditions. The mean response times were 507.7 ± 82.9 ms and 484.0 ± 87.8 ms for the first and second sessions, respectively. There was no difference in the task performance of both sessions across the conditions.

#### Statistical Modeling

**FIGURE 4:**
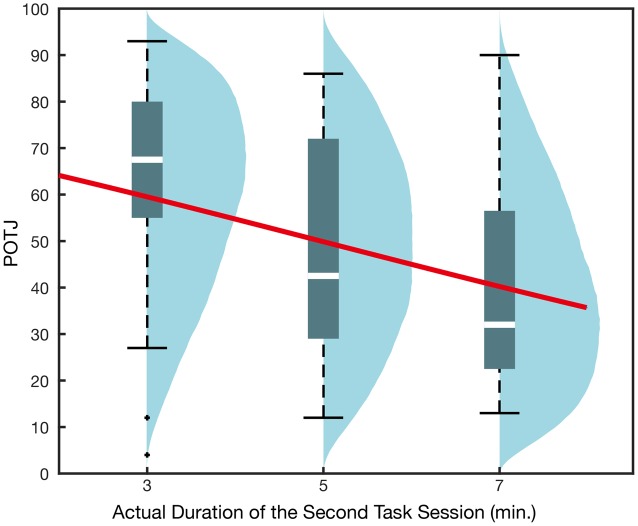
**Passage of time judgment in Experiment 2.** The *x*-axis corresponds to the durations of the second task session. All participants were instructed that the two task sessions would take 5-min each and underwent the first task session, whose duration was actually 5 min. The overlaid regression curve indicates mean q_i_ obtained in GLMM recruiting the duration of the second session only as the explanatory variable. Blue density plots are simulated POTJ distribution density incorporating random effects.

The GLMM using actual duration alone as an explanatory variable yielded a negative regression coefficient whose 95% confidence interval did not include 0 (**Table 5**, **Figure 4**). That is, as in Experiments 1a and 1b, task durations longer than the instruction led to slower POTJ, while shorter durations resulted in faster POTJ. The model selected based on AIC indicated that higher boredom and task difficulty, as well as task duration, predict slower POTJ (**Table 6**). The valence and significance of regression coefficients remained almost the same when we used linear modeling assuming normality instead of binomial GLMM (Intercept: β = 100, *p* < 10^-11^; Duration: β = -5.6, *p* = 2.6 × 10^-3^; Boredom: β = -0.41, *p* = 3.8 × 10^-4^; Difficulty: β = -0.19, *p* = 0.23). No significant correlations among the variables were found and adding sex as an explanatory variable did not improve AIC.

**Table 5:** Generalized linear mixed model recruiting task duration only as an explanatory variable for the results of Experiment 2.

	β	*SE*	*Z*	P (>|Z|)
Intercept	1.2	0.50	2.4	1.9 × 10^-2^
Duration (min.)	-0.23	0.09	-2.5	1.3 × 10^-2^

	**σ**	***SE***		

Random effect	1.2	0.11		


*AIC = 260.4, conditional R^2^ = 0.31; *SE*, standard error; Z = Wald statistic; P(>|Z|) = probability that a more extreme estimation of β than observed is obtained given that true β = 0; GLMM, generalized linear mixed model.*

**Table 6:** Generalized linear mixed model selected based on AIC for the results of Experiment 2.

	β	*SE*	*Z*	P (>|Z|)
Intercept	2.4	0.53	4.6	4.3 × 10^-6^
Duration (min.)	-0.25	0.08	-3.0	2.6 × 10^-3^
Boredom	-0.02	5.0 × 10^-3^	-4.1	4.5 × 10^-5^
Difficulty	-0.01	7.5 × 10^-3^	-1.6	0.12

	**σ**	***SE***		

Random effect	1.0	0.09		


*AIC = 247.5; conditional *R*^*2*^ = 0.32; SE, standard error; Z = Wald statistic; P(>|Z|) = probability that a more extreme estimation of β than observed is obtained given that true β = 0; GLMM, generalized linear mixed model; AIC, Akaike information criterion.*

### Discussion

First and most importantly, the size of the random effect obtained in Experiment 2 was comparable to Experiments 1a and 1b. Therefore, the relatively large random effect, which is persistently observed in the experiments so far, is likely to have reflected the variance of duration estimation *per se*, rather than individual differences in prior temporal expectations.

As for the relationships between POTJ and duration, the same tendency that the task duration lasted longer than expected predicts slower POTJ was observed again, supporting our first hypothesis that POTJ would be proportional to the discrepancy between expected and felt durations of an event. In addition, the overall level of POTJ rating was lower than it was in Experiment 1a, which is equally likely due to the reduced attentional demand or the number of the sessions. That is, the use of an easier task might have mitigated an underestimation of the task duration by directing participants’ attention to the task duration, thus leading to a slower POTJ. Or, alternatively, participants’ expectation on how 5 min should feel like might have been calibrated according to the task in the first session, thus leading to a slower POTJ compared to the previous two experiments.

## Experiment 3

**FIGURE 5:**
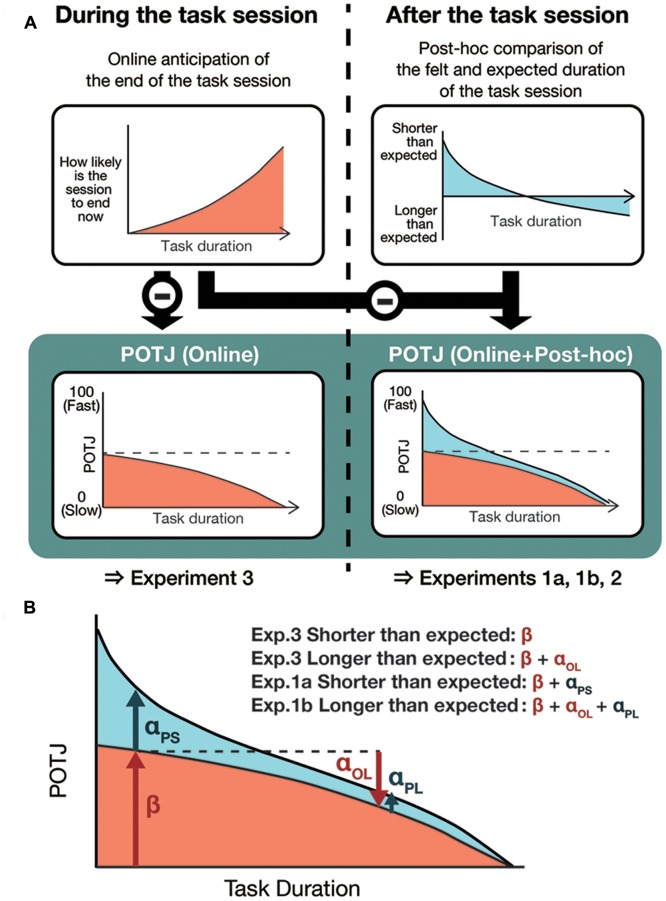
**Schematics of the hypothesis to be tested in Experiment 3.** Our hypothesis assumes two distinct components underlying POTJ, namely, *post hoc* and online ones. While the *post hoc* component can contribute to both fast and slow POTJs, the online component only decreases POTJ, which is a logical consequence of the asymmetry of temporal experiences. **(A)** Therefore, according to the hypothesis, during task sessions, one will never report fast POTJ yet will report slow POTJ when the duration of the session exceeds the prior expectation. **(B)** An illustration of the statistical model. α*_OL_* stands for the effect of the longer than expected session duration on the online component. α*_PS_* and α*_PL_* indicate effects of the *post hoc* comparison in the shorter- and longer-than-expected conditions respectively. *β*, intercept, at the same time represents the online component in the shorter-than-expected condition.

Experiment 3 was conducted to evaluate our second hypothesis that there are two distinct components that POTJ is based on: *post hoc* comparison between felt and expected duration of an event and online anticipation of the end of the event (**Figure 5A**). Note that these two components contribute to POTJ differently. That is, while the *post hoc* component can engender fast or slow POTJ when the felt duration of an event was shorter or longer than expected, respectively, the online components cannot contribute to faster POTJ. This is a necessary consequence of the asymmetry of temporal experiences that one can never know that an ongoing event is “shorter than expected” before it ends. Therefore, to dissociate *post hoc* and online components of POTJ, in Experiment 3, we asked participants to rate POTJ in the middle of the task session unexpectedly, instead of filling in the questionnaire after the session. By doing so, we expected to eliminate the *post hoc* component and extract the online component only.

### Methods

Twenty four males and fifteen females (age range = 18-25 years, mean age = 19.87 ± 1.61) participated in Experiment 3. None of the participants participated in Experiments 1a, 1b, and 2. The apparatus was identical to the other experiments. Note that there were other five participants who were excluded due to technical failure and misunderstanding of the instructions.

#### Task and Procedure

The task was the 2-back task; the same used in Experiments 1 and 2. The participants were assigned to either a shorter- or longer-than expected condition. The numbers of participants in the shorter- and longer-than expected condition were 20 and 19, respectively. The alleged duration of the tasks was 5 and 3 min, respectively, in each condition. Prior to the task session, participants completed a task-irrelevant filler questionnaire and familiarized themselves with VAS. After practicing the task briefly, participants entered into the chamber and started to perform the task. Three (in the shorter-than-expected condition) or six (in the longer-than-expected condition) minutes after the beginning of the task, a VAS asking POTJ unexpectedly showed up on the monitor and participants responded by a mouse click (for the illustration of the time course, see **Figure 1**). The query shown on the screen was slightly modified as follows: You are still on the way and here is a question. During the task so far, how fast the time seemed to be passing by? This modification was included to prevent participants from mistaking the sudden appearance of the question for the end of the session. Not that the actual question was in Japanese. Following participants’ response, 2-back task was resumed and continued until 1 min after the appearance of VAS. After the task session, participants completed another questionnaire that assessed the same affective variables that were measured in Experiments 1a, 1b, and 2. We additionally asked participants if they understood the sudden question during the task session and answered it as intended after completion of the questionnaire.

#### Analysis

Results from the 3 min condition in Experiment 1a and the 3 min condition in Experiment 1b were reincorporated, respectively, in the analysis as shorter-than-expected and longer-than-expected conditions with the *post hoc* component. GLMM was again recruited and the online and *post hoc* components of POTJ were separately modeled as categorical variables:

$$\begin{array}{c} \text{POTJ}∼\text{Binomial}\left(q_{i},100\right)\\ \text{where logit}\left(q_{i}\right)=\beta +\alpha_{OL}+\alpha_{PS}+\alpha_{PL}+r_{i}\\ r_{i}∼N\left(0,\sigma^{2}\right) \end{array}$$

Here, β indicates intercept and α*_OL_* stands for the effect of longer than expected session duration on online POTJ. α*_PS_* and α*_PL_* indicate effects of the *post hoc* component in shorter-than-expected and longer-than-expected conditions respectively. r_i_ is the random effect. The model is illustrated in **Figure 5B**.

### Results

**FIGURE 6:**
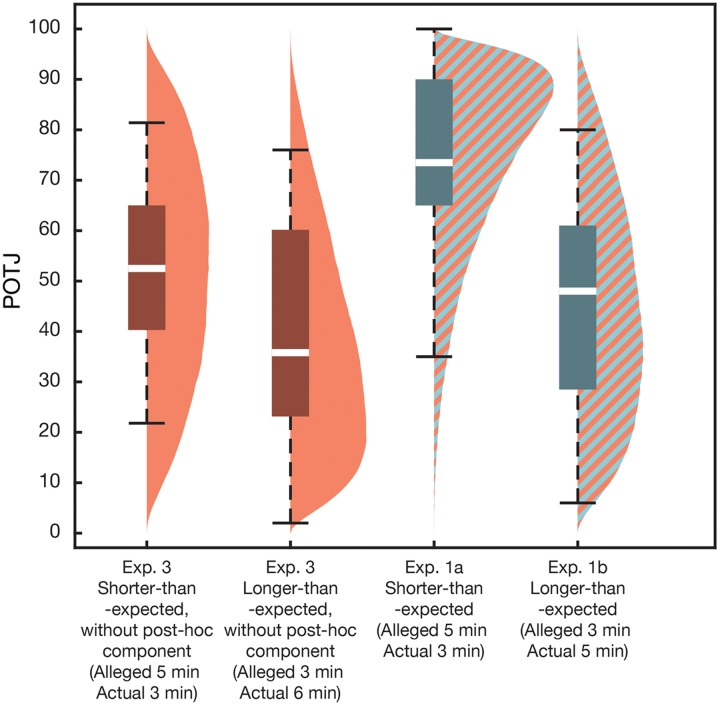
**Passage of time judgment in Experiment 3.** Eliminating the *post hoc* component on POTJ significantly slowed POTJ in the shorter-than-expected conditions (see 1st and 3rd columns), while it did not affect POTJ much in the longer-than-expected conditions (see 2nd and 4th columns). In addition, even without the *post hoc* component, longer-than-expected task durations gave rise to a slower POTJ than usual (compare 1st and 2nd columns).

The results are shown in **Figure 6** and **Table 7**. A significant effect of the *post hoc* component was found only in shorter-than-expected conditions. The effect of the duration being longer than expected on the online component of POTJ was also observed. The valence and significance of the regressors remained almost the same when we used linear modeling assuming normality instead of binomial GLMM (Intercept: β = 52, *p* < 10^-15^; α*_OL_* = -14, *p* = .06; α*_PS_* = 22, *p* = 3.4 × 10^-3^; α*_PL_* = 6.5, *p* = 0.36). Additionally, inspection on data of participants in Experiment 3 revealed some significant correlations among the variables (POTJ and boredom, *r* = -0.57, *p* = 0.008; arousal and correct response rate, *r* = 0.48, *p* = 0.03). Adding sex as an explanatory variable did not improve AIC.

**Table 7:** Online and *post hoc* components of POTJ modeled by GLMM in Experiment 3.

	Estimated effect	*SE*	*Z*	P (>|Z|)
*β*	0.08	0.24	0.34	0.73
α*_OL_*	-0.76	0.35	-2.2	0.03
α*_PS_*	1.2	0.36	3.5	5.2 × 10^-4^
α*_PL_*	0.39	0.37	1.1	0.29

	**σ**	***SE***		

Random effect	1.0	0.09		


*AIC = 310.6; conditional *R*^*2*^ = 0.34; SE, standard error; Z = Wald statistic; P(>|Z|) = probability that a more extreme estimation of β and α than observed is obtained given that true β or α = 0; GLMM, generalized linear mixed model.*

### Discussion

Experiment 3 revealed that (1) eliminating the *post hoc* component eliminates fast POTJ; however, it does not affect slow POTJ much and (2) the session duration lasting longer than expected led to slow POTJ in an online manner. These results support our second hypothesis that POTJ is based on online and *post hoc* components, as illustrated in **Figure 5**. Note that the slight duration difference between two longer-than-expected conditions is ignored here, although we do not believe it impairs validity of our hypothesis as shown above.

One possible concern is that VAS on the monitor worked as a certain time cue and affected participants’ temporal expectation. Although the appearance of VAS was totally unexpectable and VAS did not contain any explicit temporal information, it is possible that participants interpreted that it was at midmost (i.e., 2.5 or 1.5 min). If this is the case, relatively slower POTJ in shorter-than-expected condition in Experiment 3 might be explained by discrepancy between the actual task duration at the time point of VAS (3 min) and implied duration (a half of the instructed duration = 2.5 min), without assuming separate online and *post hoc* components. Although it is technically impossible to exclude this possibility from the present data only, there are several reasons that this alternative interpretation is implausible. Firstly, in informal questions after the experimental procedures were completed, most participants stated that they did not have any idea how many minutes had passed at the time point of the sudden question. In addition, it is not likely that difference between expected 2.5 min and felt 3 min lead to median POTJ as low as 50, considering that median POTJ for the 7 min condition in Experiment 1a was above 60 (**Figure 2**).

## General Discussion

We showed that (1) average POTJ is a function of the difference between expected and actual duration of a task, (2) that the large individual differences of POTJ are likely due to a relatively large variance of duration estimation, and (3) online and *post hoc* components contribute to POTJ. In addition, several variables other than the violation of temporal expectation (e.g., sadness, arousal, boredom, task difficulty and correct rate) also predicted POTJ, although the results were inconsistent across experiments with the exception boredom. In light of our current model, it is natural to interpret that those variables affected POTJ by distorting duration estimation, considering previous studies that reported links between various affective states and duration perception (Gil and Droit-Volet, 2011; Wackermann et al., 2014).

The current model of POTJ seems to explain our daily experience of the “speed of time” well. For example, oft-expressed “time flies when you are having fun” seems to reflect discrepancy between temporal expectations based on external time cues and shortened internal duration estimation due to engaging activities. Note that some reported an opposite causation that people attribute their felt time quickening to their enjoyment (Sackett et al., 2010). Another example is saccadic chronostasis (Yarrow et al., 2001) in daily contexts. When you make a saccade onto a clock, you strongly feel that time is slowed down or even stopped, as its name suggests. In contrast, while every saccade accompanies post-saccadic time dilation, people do not experience slow POTJ each time they make a saccade. This is likely because people have exceptionally precise temporal expectation about intervals between ticks of clocks, while they usually do not have such expectations for other visual events.

One important implication of our current model is that POTJ is always “about” or “defined in relation to” certain external events. This is a necessary consequence of framing POTJ as based on discrepancy between expected and estimated durations of events. In contrast, previous studies in everyday life situations, participants were asked to report POTJ at a certain moment (Droit-Volet and Wearden, 2016; Droit-volet et al., 2017). We suspect that participants in those studies interpreted such instructions differently depending on experimental settings. For example, in conditions where participants were asked to estimate brief durations and then report POTJ, it is likely that they answered POTJ about activities they were engaging into at the moment of the alert, rather than the durations they estimated. This accounts for the lack of correlation between estimated durations and POTJ in the conditions, since the authors did not control participants’ temporal expectations about daily events. In contrast, in the second experiment in Droit-volet et al. (2017), participants were frequently (once in 10 to 17 min) asked to estimate long (2–8 min) durations without watching clocks. In such a situation, experimental events are likely to have dominated their daily lives, and as a result, passage of time was judged in relation to experimental stimuli to be timed, naturally correlating with estimated durations.

Lastly, although the present study demonstrated that POTJ follows discrepancy between expected and estimated durations, detailed natures of temporal expectation and estimation are not necessarily clear. Future studies should address, for example, contributions of memory related temporal information to POTJ, which underlies retrospective duration judgments, or temporal expectations at different time scales.

## Ethics Statement

This study was carried out in accordance with the recommendations of the institutional review boards of The University of Tokyo, with written informed consent from all subjects. All subjects gave written informed consent in accordance with the Declaration of Helsinki. The protocol was approved by the institutional review boards of The University of Tokyo.

## Author Contributions

RT and YY designed the study. RT conducted the experiments. RT and YY wrote the paper.

## Conflict of Interest Statement

The authors declare that the research was conducted in the absence of any commercial or financial relationships that could be construed as a potential conflict of interest.
